# Perceived Suicide Stigma and Associated Factors in Chinese College Students: Translation and Validation of the Stigma of Suicide Attempt Scale and the Stigma of Suicide and Suicide Survivors Scale

**DOI:** 10.3390/ijerph18073400

**Published:** 2021-03-25

**Authors:** Yang Wu, Zhenzhen Chen, Philip J. Batterham, Jin Han

**Affiliations:** 1 School of Marxism, Huazhong University of Science and Technology, Wuhan 430074, China; 2 Research Center for Educational Neuroscience, School of Educational Science, Huazhong University of Science and Technology, Wuhan 430074, China; 3 Research Center for Social Psychology, Central China Normal University, Wuhan 430079, China; 4 College of Psychology, Central China Normal University, Wuhan 430079, China; czzpsych06@foxmail.com; 5 Centre for Mental Health Research, The Australian National University, Canberra 2601, Australia; philip.batterham@anu.edu.au; 6 Black Dog Institute, The University of New South Wales, Sydney 2031, Australia

**Keywords:** perceived suicide stigma, STOSA, STOSASS, SOSS, China

## Abstract

This study aims to translate and validate two perceived suicide stigma scales, including the Stigma of Suicide Attempt Scale (STOSA) and the Stigma of Suicide and Suicide Survivor Scale (STOSASS) into Chinese language, examining the factor structure, and assessing the correlation between suicide stigma and a series of variables. After translating and back translating the STOSA and STOSASS, an online survey was administrated to 412 college students in China. These two scales were tested for their dimensionality in a series of confirmatory factor analyses. A series of regression analyses were conducted to examine the factors that are associated with perceived and public suicide stigma, including demographics, psychological distress, suicidality, suicide exposure, and perceived entitativity of suicide ideators, decedents, and survivors. The results showed that the two translated scales, STOSA and STOSASS, were reliable (Cronbach’s α = 0.79~0.83) and valid in Chinese contexts and it can be treated as unidimensional scales. Suicidality, exposure to suicide, and perceived entitativity of suicide-related persons were significantly associated with higher endorsement of public suicide stigma (SOSS Stigma, *p* < 0.03), but not perceived stigma (STOSA, STOSASS). Higher levels of psychological distress were associated with both higher perceived and public suicide stigma (*p* < 0.05).

## 1. Introduction

Suicide is a severe public health threat that claims nearly a million deaths per year worldwide, and it is also the second leading cause of death in young people that are aged between 15 and 29 years [1]. In China, suicide is the leading unnatural cause of deaths in college students [2]. Increasing evidence suggests that socio-cultural factors, such as social beliefs or stigmas about suicide, may contribute to suicide [3,4,5,6,7,8].

Suicide stigma refers to a social process involving overgeneralized perceptions, negative attitudes, and discriminatory behavioral intentions and acts towards people who are suicidal and people bereaved by suicide [9,10]. Suicide stigma was found to be associated with low help-seeking intentions [11,12,13] and behaviors [14], increased psychological distress [10], and higher suicide risk [15]. Suicide stigma may also contribute to underreporting of suicide death [3,8], as well as reducing people’s willingness to participate in suicide prevention programs [16]. While there have been discussions on the impact of Chinese cultural beliefs on suicide stigma [17], there is still limited empirical research on suicide stigma in the Chinese population. The lack of validated measurements is one possible reason.

### 1.1. Suicide Stigma and Perceived Suicide Stigma

Theoretical and empirical research has differentiated several distinct yet interconnected aspects of suicide stigma, including public stigma, self-stigma, and perceived stigma.

*Public stigma* (also known as personal stigma) refers to “the phenomenon of large social groups endorsing negative stereotypes about and acting against a stigmatized group” [18]. Key to the measurement of public stigma is people’s endorsement of stigma, i.e., people’s agreement with the stigmatized attitudes towards the target group. The overall endorsement of suicide stigma in a target population is often used as a measure of the prevalence of suicide stigma in that population. A high and society-wide endorsement of suicide stigma suggests the existence of public stigma against suicide. Amongst individuals with lived experience of suicidal thoughts and/or behaviors, a higher endorsement of suicide stigma usually reflects more negative attitudes that are internalized towards themselves, known as *self-stigma* [19].

*Perceived stigma*, on the other hand, relates to people’s beliefs about, and perceptions of, others’ stigmatizing attitudes towards individuals with suicidal thoughts and/or behaviors, regardless of whether they personally endorse those attitudes [20,21]. Higher perceived suicide stigma among healthy people may predispose them to be less likely to seek help when they experience suicide ideation in the future, as preliminary evidence showed that higher perceived suicide stigma was indeed associated with decreased help seeking behaviors in Latino immigrants [12] and Dutch and Flemish populations [22]. Because the effect of perceived stigma resides in expected social pressure [22], people from collective cultures, such as Latino, North European, and East Asian cultures may be especially susceptible to perceived suicide stigma [23,24].

### 1.2. Measurement of Suicide Stigma

Several scales of suicide stigma, including public stigma, self-stigma/internalized stigma, and perceived stigma, have been developed in the past decade [9,21,25,26]. The Stigma of Suicide Scale (SOSS) that was developed by Batterham et al. [25] is one of the most popular measures capturing public stigma. The SOSS has been translated and validated in different languages, including Arabic [27], Japanese [28], and Chinese [29]. The scale has been shown to predict professional help-seeking [13,30], as a higher score on the SOSS was associated with lower help-seeking intentions [30].

Perceived suicide stigma can be measured by the Stigma of Suicide Attempt Scale (STOSA) and the Stigma of Suicide and Suicide Survivor Scale (STOSASS) [21]. STOSA focuses on attempted suicide, while the STOSASS mainly evaluates stigma towards suicide death. Both of the scales are reliable [21] and widely used in Western samples [31,32,33]. Recently, Han et al. [29] translated and validated the short-form of SOSS in Chinese, which chiefly assesses respondents’ endorsement of suicide stigma, but not the perception of suicide stigma [34]. Given the distinct significance of perceived stigma on suicide prevention, it is important to translate STOSA and STOSASS and validate them in Chinese samples, examining their relationship with public stigma and help seeking in a Chinese context.

### 1.3. Factors Associated with Suicide Stigma

The current literature suggests a few potential factors that are associated suicide stigma [10,14], including exposure to suicide-related information (suicide exposure), the severity of mental health symptoms, and perceived entitativity of suicide decedents (i.e., the extent to which a group of people were perceived as an entity).

First, individual exposure to suicide-related information or events may alter the levels of suicide stigma by influencing one’s knowledge of suicide and the awareness of the public attitudes towards suicide. Here, the suicide exposure was defined as a broad contact with suicide related information or events, including engaging with suicide in a movie/TV show, workplace, or personal life (also see Cerel et al. [35]). A higher level of exposure, such as experiencing the suicide of a loved one, may raise people’s awareness of the existing public stigma towards suicide. A study among 3432 bereaved adults in the UK showed that previous experience of suicide bereavement increased their perceived stigma of suicide, which may lead to a higher risk of suicide ideation [36]. At the same time, being continually exposed to media depictions of suicide may increase one’s endorsement of suicide myths and stigmatizing attitudes specific to cultures [37]. On the other hand, lower exposure to suicide is often associated with poorer suicide literacy [38], which has been found to be associated with stigmatizing views of people who attempt or die by suicide [38]. Therefore, while personal exposure to suicide may increase perceived stigma, its relationship with public stigma is complicated, which warrants further investigation.

Second, the severity levels of suicidality and psychological distress may also be associated with suicide stigma. Suicidality represents a wide range of suicide-related cognitions, emotions, and behaviors [39], including suicidal ideation, planning, and attempts. A large body of evidence suggests that suicide stigma is linked with suicidality and psychological stress [40,41,42]. A recent large-scale survey of college students in the U.S. revealed a significant association between perceived stigma and greater odds of suicide ideation, planning, and attempt [15]. However, most of the previous studies had only examined either perceived or public stigma (not both), and they were conducted in Western countries. To date, few studies have compared the difference in the impacts of suicidality and psychological distress on public and perceived suicide stigma.

Finally, the perceived entitativity of suicide decedents is a new emerging potential factor that is associated with suicide stigma. Previous studies suggest that people tend to perceive various forms of social groups along a continuum of entitativity [43,44], which is, the extent to which an aggregate of the group members can be perceived as having “the nature of an entity, of having real existence” [43]. Social groups with high entitativity are more likely to be seen as a coherent unit (e.g., members of a professional sports team); while social groups with low entitativity are most likely groups with highly heterogenous members being treated as a whole merely by chance (e.g., people in a queue in a supermarket). Key components that determine perceived group entitativity include homogeneity in physical features and goals, member interactivity, clear group boundaries, a common history [45], and a presumed underlying “essence” [46,47,48].

Research on entitativity has documented a variety of ways in which entitativity affects group-based information processing [49,50,51,52]. For example, Crawford, Sherman, and Hamilton [53] showed that high entitativity would facilitate transference of traits from one member to another and, therefore, individual group members are more likely to be evaluated in terms of the stereotypic qualities expected of the group. A corollary of this is that only when a group is perceived as a “unit” with its members sharing at least some similarities could bias against the group arise [54]. Because intergroup bias and stereotypes are integral parts of stigma, a higher perceived entitativity of a group may precondition the formation of stigmatized attitudes towards the group. Kende and McGarty [55] recently formulated a contingency model about stigma and prejudice expression, in which entitativity featured prominently as a key node in determining the stigma expression. There is also evidence showing that perceived entitativity could affect the responses to stigma among persons with mental illness [47,56]. Therefore, it is important to investigate the extent to which perceived entitativity of suicidal individuals may impact the levels of suicide stigma.

### 1.4. Method Effects and Its Influence on the Factorial Structure of STOSA and STOSASS

Although STOSA and STOSASS were initially developed as unidimensional scales with positively and negatively worded items [21], in exploratory factor analysis both of the scales turned out to be bidimensional and the two types of items converged towards two separate factors [21]. This phenomenon happens in several other scales with similar structure [57,58].

This inconsistency indicates that individual responses may have systematically differed based on the direction of item wording, a phenomenon known as method bias. It could confound the meaning of the sum and interpretation of the scores. Various factors, including careless responding [59] and acquiescence bias [60], could contribute to the “response tendencies that raters apply across measures, similarities in item structure or wording that induce similar responses, the proximity of items in an instrument, and similarities in the medium, timing, or location in which measures are collected” [61]. Researchers often employ the multitrait-multimethod (MTMM) conceptual framework to decompose the substantive and method components among items on a scale [60,62].

Under this framework, two broad subtypes of model could be specified [60], including the *correlated trait*, *correlated methods* (CTCM) model and *correlated trait*, *correlated uniqueness* (CTCU) model, as well as their many variants. Both types of model have its own merits and limits [63], yet specified together on a same dataset, they could serve to decompose the total variances of the data into variances that are attributable to traits and methods. In general, should the CTCU or CTCM models fit data better than baseline models, one could conclude that the method bias does exist, which, in turn, would suggest that the bidimensional model that is generated by EFA is merely a result of measurement artifacts and the theoretically unidimensional model should be preferred.

### 1.5. The Current Study

Most previous studies have examined perceived and public suicide stigma separately in Western contexts, and there is limited investigation of the role of entitativity in suicide stigma. The aim of the present study, therefore, is (a) to translate and validate STOSA and STOSASS in Chinese contexts, and, during the process, (b) to compare the factors that are associated with perceived and public suicide stigma in a Chinese college sample. Both perceived and public suicide stigma were measured, together with demographic variables, exposure to suicide, psychological distress, suicidality, and perceived entitativity of suicide. We hypothesize that psychological distress, suicidality, and suicide exposure are positively associated with perceived and public suicide stigma.

## 2. Method

### 2.1. Participants

A sample of 499 students (260 males, 52.10%) from a public university in central China participated in the study for either course credits or a small gift (approx. 5 yuan, equivalent of 0.71 USD). The participants were mainly first year students attending a general education course (Morality and Basics of Law) recruited either by email or classroom invitation. The participants with incomplete answers on demographics were excluded from the regression analysis (N = 87, 17.4%), but they were retained in factorial analysis. The excluded cases did not differ significantly with included cases on key variables, including STOSA, STOSASS, and SOSS. The final data for our main regression analysis consisted of 412 participants (220 males, 53.40%). The mean age was 18.86 years (*SD* = 0.39). Table 1 provides detailed demographic information.

### 2.2. Translation

The English version of Stigma of Suicide Attempt Scale (STOSA) and the Stigma of Suicide and Suicide Survivor Scale (STOSASS) were translated by WY and JH into simplified Chinese, with written authorization from the author, Scocco. YYS back-translated the Chinese version into English, which was checked by Scocco for semantic equivalence. WY and JH then discussed the significant disparities in both versions, revising until a faithful translation was achieved.

In order to further ensure translation quality, we compared the functional correspondence between of the original and translated versions, as in Han et al. [29]. Another seventy-nine students majoring in English Language Study were recruited to complete both English and Chinese versions of the scales. All of them had credentialed English proficiency and they were invited by their teachers to participate on a voluntary basis. The order of the two language versions was counterbalanced. Three participants accepted the researcher’s invitation for a follow-up interview about the semantic and tonal equivalence of the items. All 79 participants were informed about their right to quit at any time and they were given a small gift of one yuan (approx. 0.14 USD) at the end of the study.

### 2.3. Measures

#### 2.3.1. Suicide Stigma

The data and measures were a part of a large survey on “Mental health profile and health-related attitudes of Chinese participants”. Aside from the measures as reported below, several other scales were also included in the survey, such as self-efficacy, help-seeking, and measures on cognitive styles. Because they were unrelated to the aim of this study, we did not report them in the current study.

The Stigma of Suicide Attempt Scale (STOSA) is a 12-item scale assessing respondents’ perceptions of general social attitudes towards suicide attempters, with higher scores indicating stronger stigmatizing attitudes. Half of the twelve items are negatively worded (item 1, 2, 3, 4, 8, 10). In the current study, the participants rated each item on a seven-point Likert scale, ranging from 1 (“*strongly disagree*”) to 7 (“*strongly agree*”). It showed good reliability in the past (lamda-2 = 0.76 for general population [21]). In the present study, Cronbach’s α = 0.79.

The Stigma of Suicide and Suicide Survivor Scale (STOSASS) shares similar item content with STOSA, with the target of each item focusing instead on people who died by suicide and people bereaved by suicide. Three items in the STOSASS are repeated in two versions, the first (version a) referring to people who died by suicide, and the second (version b) referring to people bereaved by suicide. The two versions share nine identical items and each have three separate items. The participants rated each item on a seven-point Likert scale, ranging from 1 (“*strongly disagree*”) to 7 (“*strongly agree*”). It showed good reliability in the past (Cronbach’s α = 0.88) [33]. In the present study, Cronbach’s α =0.80 (decedents) and 0.83 (survivors), respectively.

The short-form of Stigma of Suicide Scale (SOSS) Chinese Version is a 12-item scale translated and validated by Han et al. [29] on the basis of its original version developed by Batterham et al. [25]. The participants rated a typical person who has died by suicide on 12 adjectives, which has been shown to be divided into three dimensions, Stigma, Isolation, and Glorification. SOSS uses a five-point Likert scale ranging from 1 (“*strongly disagree*”) to 5 (“*strongly agree*”). In the current study, Cronbach’s α for the three dimensions were, 0.81 (glorification), 0.82 (Isolation), and 0.77 (Stigma), respectively.

#### 2.3.2. Other Measures

A single item from the item bank of Rapid Measurement Toolkit for Suicidality were used to measure respondents’ suicidality [64]. The item is “I wanted to make an active suicide attempt”. The participants rated on a five-point Likert scale ranging from 1 (“*strongly disagree*”) to 5 (“*strongly agree*”).

The Kessler Psychological Distress Scale (K6) was used to measure respondents’ level of psychological distress [65]. The Chinese version was translated and validated in the World Mental Health Survey [66]. The participants rated the six items of K6, using a five-point Likert scale ranging from 1 (“*strongly disagree*”) to 5 (“*strongly agree*”). Cronbach’s α is 0.90.

Exposure to suicide was assessed by a 10-item multiple choice scale that was adapted from the Level of Contact Report [67], and the adapted version has been used in past research on suicide stigma [38]. The Chinese version was translated and validated by Han [68]. Each of the 10 items depicted a situation with a specific level of exposure to suicide, and the participants responded by checking all of the situations that they had experienced. The level of exposure ranges from 0 to 9, with the higher score meaning higher level of exposure. The ten levels are: no exposure (0), observing suicide in a movie or television show: (1) documentary on suicide (2), colleague attempted or died by suicide (3), provided services to someone who attempted or died by suicide (4), acquaintance attempted or died by suicide(5), relative attempted or died by suicide (6), close friend attempted or died by suicide (7), lived with someone who attempted or died by suicide (8), or respondent attempted suicide (9). The participant’s final score on the scale is the maximum level among all of the items that they endorsed.

Entitativity was assessed by a scale that was developed by Lickel et al. [44] and it was adapted to the study’s focus on suicide. Participants were first introduced to the concept of entitativity by reading a short instruction. The participants then were asked to rate three targets, “suicide decedents”, “suicide survivors”, and “suicide ideators”, along a nine-point Likert scale ranging from 1 (not a group at all) to 9 (very much a group). Ten filler groups (e.g., people in line at the bank; Polish citizens; members of a family) varying greatly in entitativity were also included to provide reference for participants.

Two items were added to screen clearly careless responders, e.g., I have been to all the countries in the world [69].

Demographics were measured by multiple choice items, including gender, relationship status, sexual orientation, parents’ educational level, and college majors. Table 1 presents choice options for each item.

### 2.4. Procedure and Analysis

The participants were invited to participate in an online survey. The order of all items within each scale was randomized. After completing the main section of the survey, participants answered demographic questions, including gender, age, relationship status, major, sexual orientation, and they were offered a token for course credits or a small gift. This study was carried out in accordance with the recommendations of the American Psychological Association ethical guidelines. The study was approved by Huazhong University of Science and Technology and it was carried out according to the guidelines established by the Helsinki Declaration. At the beginning of the survey, a short description of the study informed all participants of their right to choose to participate and the right to withdraw from the study at any time. The participants proceeded to survey only after clicking consent.

We tested the content convergence English and Chinese versions of STOSA and STOSASS in the sample of seventy-nine English major students; the data were analyzed using Pearson product-moments correlation and paired-sample t-test between the two versions.

For the main study, the data of STOSA and STOSASS were first analyzed by confirmatory factor analysis. A total of nine models following MTMM framework were tested two accommodate the possible method bias. The CFA was conducted in Lisrel 8.80, using a maximum likelihood (ML) estimation procedure on a covariance matrix. The latent factor variances were fixed at one to set the scale.

Among the nines models tested, Model 1 was the theoretically conceived unidimensional model, and Model 2 was the empirically derived bidimensional model with two substantive factors, one for positively worded items and another for negatively worded items [21]. Model 3–5 belonged to the category of CTCU models. In Model 3 and 4, a substantive factor for stigma was specified, and the residuals of positively worded items were freed to intercorrelate in Model 3; likewise, the residuals of negatively worded items in Model 4. Model 5 specified correlated uniqueness within items while using the same wording method, and residual intercorrelations across wording methods were forbidden. Model 6–9 belonged to the CTCM models, in which two sets of factors, substantive factors and method factors, were specified and the cross-set correlation of factors were always set to zero. Model 6 specified a substantive factor for suicide stigma and a method factor for positively worded items, whereas Model 7 a method factor for negatively worded items. Model 8 simultaneously specified two correlated method factors for both types of items. Based on Model 8, Model 9 set the correlation between two method factors to zero. Because two subversions existed in STOSASS, all nine models were tested in each subversion.

The goodness-of-fit indices used in model comparison included: (1) Chi-square; (2) Root mean square error approximation (RMSEA); (3) the standardized root mean square residual (SRMR); (4) Akaike’s information criterion (AIC); (5) Nonnormed fit index (NNFI), (6) the goodness-of-fit index (GFI), and (7) the comparative fit index (CFI).

A combined use of these indices is necessary to adequately evaluate a model, given their respective strengths and weaknesses [70,71,72,73]. For NNFI, CFI, and GFI, the value criterion for an acceptable fit is 0.90 and for a better fit is 0.95. For RMSEA, the criterion for a good fit is 0.06, the smaller the value, the better the fit. A criterion of 0.08 for adequate fit (small value indicates better fit) was often suggested for SRMR [71]. Because SRMR does not penalize complex models, indices that penalize complex models, such as NNFI and RMSEA, could help to interpret values of SRMR. Finally, AIC is important in model comparison contexts, especially for non-nested alternative models that are unsuitable for χ^2^ tests. In a series of models, the one with a smaller value of AIC suggests a better fit.

The mean scores of all main variables in the studies were tested for differences across gender, relationship status, sexual orientation, parents’ educational level, and college major, using analysis of variance (ANOVA) or an independent t test. For significant results of ANOVA, we conducted pairwise comparisons by Tukey’s HSD tests. Regression analysis was conducted to test the different relationships of stigma endorsement and stigma perception with K6, suicide exposure, suicidality, and perceived entitativity of suicide ideators, decedents, and survivors. We tested a total of six regression models with STOSA, STOSASS (two subscales), and SOSS (three subscales) as the outcome variables. In each model, we simultaneously included as independent variables the K6, exposure to suicide, suicidality and entitativity measures, as well as the demographic variables. Gender, parent educational level, sex orientation, relationship status, and major were all categorical variables and, thus, dummy coded. The reference groups for each categorical variable were gender (female), parent educational level (only one have higher education), sex orientation (non-heterosexual), relationship status (not in a relationship), and major (medicine, psychology, or related disciplines).

## 3. Results

### 3.1. Content Convergence

The sample used to test content convergence between the Chinese and the English items of STOSA and STOSASS consisted of 79 English major students (68 females), with the ages ranging from 18 to 23 years (*Mean* = 19.41, *SD* = 0.97). The results showed that items from both versions were moderately or strongly correlated (*mean* rs = 0.56), and all of the correlations were significant (*p* < 0.05).

### 3.2. Factorial Structure

All of the models were identified, except the model 5 of STOSA. Table 2(a)–(c) show the goodness-of-fit statistics. The results showed that the patterns of changes in fit indices across the nine models in STOSA, STOSASS-a, and STOSASS-b were comparable. Model 3–5 and Model 6–9 all fit data better than the theoretically unidimensional model, which suggests that method bias did exist. A closer examination of positive-wording-bias-controlling models (Model 3 and Model 6) and negative-wording-bias-controlling models (Model 4 and Model 7) as compared with the baseline Model 1 showed that the changes in fit indices were generally comparable, suggesting that the method bias from positive and negative item wording might be similar in strength. Besides, Model 5 and Model 8 were generally the best fit models, as they simultaneously controlled the item wording bias of the two directions. In general, the results suggest that the two factors in Model 2 and in Scocco et al. [21] are artifacts of method bias, and both of the scales should be treated and scored as unidimensional, rather than bi-dimensional scales.

### 3.3. Descriptive Statistics

Given that the two-factor structure of our focal scales was shown to be an artifact of method bias, we calculated their observed means based on the theoretically single-factor model and further conducted correlational and regression analyses. It is possible to examine the latent relationships among the variables, yet, given the multiplicity of models (in the current study, 6 models) to parameterize or tease out the method effect, it would be unnecessarily cumbersome to compare the minute differences among all of the possible correlation matrices obtained from the different models. Thus, we selected one of the best fitted models, Model 5, and computed latent associations between STOSA and other scales. The resulting coefficients were slightly higher in some pairs and lower in others, but the general pattern from observed scores held. Therefore, we only reported results based on the observed mean scores.

Table 1 shows descriptive statistics for Stigma of Suicide Attempt Scale (STOSA), the Stigma of Suicide and Suicide Survivor Scale-a (STOSASS-a), and the Stigma of Suicide and Suicide Survivor Scale-b (STOSASS-b). Table 3 shows the zero-order correlation between key variables. A series of t-tests and ANOVA revealed lower levels of STOSA stigma scores among participants who were in medicine, psychology, or other related disciplines. The SOSS stigma scores were significantly higher in participants who were male, heterosexual, or engineering majors, and SOSS glorification scores were significantly higher among non-heterosexual participants. No significant differences among demographic variables were found for both versions of the STOSASS and the Isolation dimension of the SOSS.

### 3.4. Regression Analysis

Table 4 shows the estimates for regression coefficients. The results showed that participants with higher levels of psychological distress tended to have higher perceived suicide stigma and public suicide stigma. Previous exposure to suicide-related information and self-reported suicidal ideation were associated with higher likelihood of glorification, but not with any other dimensions of suicide stigma or attribution to isolation. Participants with a higher tendency to view suicide decedents as an entity were more likely to endorse suicide decedents as isolated and endorse stigma. The perceived entitativity of individuals bereaved by suicide (suicide survivors) was also associated with the glorification of suicide. No significant relationship was found between the perceived entitativity and STOSA and STOSASS. The participants with a medicine, psychology, or related education background were found to have significantly lower perceived suicide stigma towards people who attempt suicide and public suicide stigma towards suicide decedents than those from an engineering background. Lower parental education levels (neither with tertiary education) were associated with higher perceived suicide stigma towards suicide attempts, but not with any dimensions of public suicide stigma.

## 4. Discussion

The primary aim of the current study was to translate and validate the Chinese versions of the Stigma of Suicide Attempt Scale (STOSA) and the Stigma of Suicide and Suicide Survivor Scale (STOSASS) in Chinese colleges students. The translated scales demonstrated reliable internal consistency and validation in the sample. These two scales are brief, hence being suitable for measuring the perceived suicide stigma in large-scale epidemiological and psycho-sociological surveys.

Although the two scales were initially conceived as unidimensional scales [21,32], past research indicates a potential bi-dimensional factor structure of the scales [21]. We explored this issue under the MTMM framework in this study. Our results indicated that the bi-dimensional structure appears to be an artifact due to method bias that is derived from reverse-coded items [61]. This finding supported the unidimensional factor structure of the STOSA and the STOSASS that was initially conceived in the original study [21].

Our results indicate that elevated psychological distress was significantly associated with public and perceived suicide stigma. This finding is consistent with previous findings in clinical samples [10,33,42]. We also found that more severe suicidality or greater exposure to suicide were associated with more glorification towards suicide decedents, but not with stigmatizing attitudes, attribution of suicide to isolation, or perceived stigma towards suicide decedents, echoing previous findings in this field [74]. The positive relationships between suicidality, suicide exposure, and glorification of suicide could be a reflection of parts of Asian cultures, where suicides have historically been interpreted as a vindication or restoration of one’s honor or as a struggle for justice [75].

Consistent with past studies on entitativity and intergroup bias [53,54,55], in the present study we found that higher scores in students’ entitativity ratings of suicide decedents and survivors were associated with greater public suicide stigma, but not with perceived suicide stigma. The effect of entitativity may relate to personality and contextual factors, such as the need for cognitive closure [46], essentialist beliefs [47], and intergroup conflicts. To better understand the underlying mechanisms that are associated with stigma, future research may benefit from integrating measures of these constructs and other aspects of social cognition. For a practical implication, the current findings on entitativity suggest that challenging stereotypes about suicidal individuals, potentially by emphasizing the diversity of people with suicidal experience, may lead to reductions in stigma.

Furthermore, we found a significant effect of education on suicide stigma. Participants with majors of medicine, psychology, or a related discipline were found to have lower public suicide stigma. High educational levels have been found to be associated with increased suicide literacy and lower suicide stigma in a few previous studies [20,29,37]. Psychoeducational programs on suicide prevention also reduced suicide stigma [12,76,77], indicating that the impact of majors on suicide stigma may be mediated by the knowledge levels of suicide, which warrants further investigation.

Several limitations of this study should be noted. First, the participants in this study were healthy college underclassmen with a relatively narrow age range, restricting the generalizability of the results. Future studies may target a more diverse sampling population, such as rural and clinical populations, to further investigate the validity of the translated scales. Second, the cross-sectional design of the study limited inferences about the direction of causality of the findings. Longitudinal research is required to understand the mechanisms and contextual elements that promote or inhibit the development of stigmatizing attitudes.

## 5. Conclusions

Despite these limitations, this study, for the first time, presented the Chinese versions of STOSA and STOSASS that could be used to assess perceived suicide stigma in the Chinese population. Both of the scales are brief, being suitable for use in large-scale epidemiological and social research. The translation of these two scales enables a more nuanced analysis of impacts of suicide stigma on suicide and suicide-related help-seeking in China, facilitating future cross-cultural comparisons of these factors. The current study also found, for the first time, that perceived entitativity of suicide decedents and survivors was associated with public suicide stigma, but not perceived stigma, which indicated the distinctiveness of these two stigma constructs.

## Figures and Tables

**Table 1:** Descriptive statistics for participants and relationship with Stigma of Suicide Attempt Scale (STOSA), Stigma of Suicide and Suicide Survivor Scale (STOSSAS), and Stigma of Suicide Scale (SOSS) scores (N = 412).

Demographic Category	N (%)	STOSA *M* (*SD*)	STOSSAS-a (Decedent) *M* (*SD*)	STOSSAS-b (Survivor) *M* (*SD*)	SOSS Glorification *M* (*SD*)	SOSS Isolation *M* (*SD*)	SOSS Stigma *M* (*SD*)
Complete sample	412 (100.0%)	3.98 (0.90)	3.69 (0.88)	3.58 (0.94)	2.07 (0.79)	3.90 (0.82)	2.55 (0.79)
**Age**							
18	51 (12.4%)	3.91 (1.09)	3.50 (1.03)	3.40 (1.02)	1.92 (0.77)	3.99 (0.88)	2.44 (0.88)
19	359 (87.1%)	3.99 (0.88)	3.72 (0.86)	3.61 (0.93)	2.09 (0.79)	3.88 (0.82)	2.57 (0.78)
Did not report	2 (0.5%)	4.13 (0.18)	3.92 (0.12)	3.88 (0.18)	2.38 (0.53)	3.83 (0.24)	2.80 (0.57)
*F*		0.18	1.37	1.18	1.27	0.36	0.74
*p*		0.84	0.26	0.31	0.28	0.70	0.48
**Gender**							
Male	220 (53.4%)	4.01 (0.87)	3.72 (0.89)	3.61 (0.95)	2.03 (0.81)	3.88 (0.87)	**2.68 (0.76)**
Female	192 (46.6%)	3.94 (0.94)	3.66 (0.88)	3.55 (0.92)	2.12 (0.76)	3.92 (0.77)	**2.41 (0.80)**
*t*		0.79	0.75	0.63	−1.12	−0.53	**3.60**
*p*		0.43	0.45	0.53	0.26	0.60	**<0.001**
**Sexual orientation**							
Heterosexual	364 (88.4%)	4.01 (0.88)	3.70 (0.87)	3.59 (0.93)	**2.04 (0.79)**	3.89 (0.83)	**2.58 (0.80)**
Non-Heterosexual	48 (11.7%)	3.76 (1.04)	3.62 (0.99)	3.50 (0.98)	**2.29 (0.74)**	3.96 (0.73)	**2.33 (0.73)**
*t*		1.54	0.55	0.60	**−2.16**	−0.62	**2.21**
*p*		0.13	0.58	0.55	**0.03**	0.54	**0.03**
**Major**							
Engineering or other related disciplines	222 (53.9%)	**4.06 (0.85) ^a^**	3.73 (0.83)	3.62 (0.88)	2.00 (0.79)	3.90 (0.83)	**2.72 (0.76) ^a^**
Liberal arts	135 (32.8%)	**3.97 (0.99) ^a^**	3.70 (0.97)	3.60 (1.01)	2.18 (0.78)	3.94 (0.81)	**2.35 (0.79) ^a^**
Medicine, psychology, or other related disciplines	55 (13.4%)	**3.67 (0.84) ^b^**	3.53 (0.86)	3.39 (0.96)	2.10 (0.76)	3.80 (0.82)	**2.39 (0.81) ^b^**
*F*		**4.12**	1.14	1.33	2.13	0.53	**11.03**
*p*		**0.02**	0.32	0.27	0.12	0.59	**<0.001**
**Relationship status**							
In a relationship	78 (18.9%)	3.96 (0.91)	3.63 (0.89)	3.52 (0.90)	2.06 (0.77)	3.86 (0.76)	2.52 (0.84)
Not in a relationship	334 (81.1%)	3.98 (0.90)	3.71 (0.88)	3.59 (0.95)	2.08 (0.79)	3.90 (0.84)	2.56 (0.78)
*t*		−0.16	−0.65	−0.63	−0.18	−0.46	−0.40
*p*		0.87	0.52	0.53	0.85	0.64	0.69
**Parental education level**							
Both with tertiary education	131 (31.8%)	**3.90 (0.96) ^a^**	3.66 (0.87)	3.57 (0.91)	2.08 (0.75)	3.83 (0.73)	2.50 (0.79)
Neither with tertiary education	217 (52.7%)	**4.08 (0.81) ^a^**	3.74 (0.84)	3.62 (0.91)	2.07 (0.81)	3.92 (0.84)	2.59 (0.80)
Only one with tertiary education	64 (15.5%)	**3.78 (1.03) ^b^**	3.61 (1.04)	3.49 (1.07)	2.08 (0.78)	3.95 (0.93)	2.54 (0.77)
*F*		**3.47**	0.64	0.48	0.01	0.71	0.46
*p*		**0.03**	0.53	0.62	0.99	0.49	0.63

Note. STOSA, Stigma of Suicide Attempt; STOSSAS, Stigma of Suicide and Suicide Survivor Scale; SOSS, Stigma of Suicide Scale. ^a, b^ For significant ANOVA tests, pairwise comparisons were conducted using Tukey’s HSD tests. Values sharing the same superscript are not significantly different. Bold values indicate *p* < 0.05.

**Table 2:** (**a**) Fit Indices of Models (STOSA); (**b**) Fit Indices of Models (STOSASS-a); and (**c**) Fit Indices of Models (STOSASS-b).

(**a**)								
**Model**	**χ^2^**	**df**	**RMSEA**	**GFI**	**CFI**	**NNFI**	**SRMR**	**AIC**
1	1228.26	54	0.27	0.60	0.66	0.59	0.21	2043.46
2	288.07	53	0.10	0.90	0.93	0.92	0.08	373.12
3	188.23	39	0.09	0.94	0.96	0.93	0.07	263.72
4	195.74	39	0.10	0.93	0.96	0.92	0.06	302.12
5	unidentified							
6	251.87	48	0.10	0.92	0.94	0.92	0.07	338.53
7	207.92	48	0.09	0.92	0.95	0.94	0.06	306.81
8	61.45	41	0.03	0.98	0.99	0.99	0.03	136.40
9	85.50	42	0.05	0.97	0.99	0.98	0.07	156.88
(**b**)								
**Model**	**χ^2^**	**df**	**RMSEA**	**GFI**	**CFI**	**NNFI**	**SRMR**	**AIC**
1	1017.60	54	0.22	0.69	0.71	0.64	0.16	1414.08
2	358.97	53	0.11	0.89	0.91	0.88	0.07	415.96
3	185.34	39	0.09	0.94	0.96	0.92	0.04	259.21
4	273.14	39	0.11	0.91	0.93	0.88	0.07	358.30
5	74.39	25	0.06	0.98	0.99	0.96	0.04	178.26
6	326.05	48	0.11	0.90	0.92	0.88	0.06	396.48
7	343.25	48	0.11	0.90	0.91	0.88	0.07	411.30
8	147.58	41	0.07	0.95	0.97	0.95	0.04	216.88
9	153.54	42	0.07	0.95	0.97	0.95	0.04	220.17
(**c**)								
**Model**	**χ^2^**	**df**	**RMSEA**	**GFI**	**CFI**	**NNFI**	**SRMR**	**AIC**
1	1005.40	54	0.23	0.67	0.76	0.71	0.17	1550.76
2	227.08	53	0.08	0.93	0.96	0.95	0.06	286.38
3	174.28	39	0.08	0.95	0.97	0.94	0.04	250.68
4	157.59	39	0.08	0.95	0.97	0.95	0.06	240.92
5	94.27	25	0.07	0.97	0.98	0.96	0.07	196.19
6	197.69	48	0.08	0.94	0.96	0.95	0.04	265.88
7	218.26	48	0.09	0.93	0.96	0.94	0.06	289.54
8	99.66	41	0.06	0.97	0.99	0.98	0.04	175.94
9	133.47	42	0.07	0.96	0.98	0.96	0.06	205.21

**Table 3:** Zero-order correlations between key variables (N = 412).

Variables	1	2	3	4	5	6	7	8	9	10	11	12
STOSA	1.00											
2. STOSASS-a	0.51 ***	1.00										
3. STOSASS-b	0.46 ***	0.97 ***	1.00									
4. SOSS-Glorification	−0.04	0.12 *	0.16 **	1.00								
5. SOSS-Stigma	0.17 ***	0.15 **	0.14 **	0.01	1.00							
6. SOSS-Isolation	0.09 ^†^	−0.04	−0.07	0.02	0.22 ***	1.00						
7. K6	0.17 **	0.15 **	0.14 **	0.23 ***	0.02	0.17 ***	1.00					
8. Suicide Exposure	−0.04	0.06	0.07	0.21 ***	−0.17 **	0.01	0.15 **	1.00				
9. Suicidality	0.04	0.13 *	0.14 **	0.27 ***	−0.10 *	0.08	0.48 ***	0.37 ***	1.00			
10. Entitativity-decedents	−0.09 ^†^	−0.04	−0.05	−0.07	0.14 **	0.18 ***	−0.25 ***	−0.14 **	−0.19 ***	1.00		
11. Entitativity-survivors	0.05	0.03	0.02	0.17 **	−0.04	0.06	0.10 *	0.04	0.03	0.03	1.00	
12. Entitativity-ideators	−0.02	−0.07	−0.08 ^†^	0.06	0.05	0.05	0.01	−0.02	−0.09 ^†^	0.21 ***	0.32 ***	1.00

Note. ^†^ *p* < 0.1, * *p* < 0.05, ** *p* < 0.01, *** *p* < 0.001.

**Table 4:** Linear regression models for suicide stigma (N = 412).

Predictors	STOSA		STOSSAS-a (Decedent)		STOSSAS-b (Survivor)		SOSS Glorification		SOSS Isolation		SOSS Stigma	
	Estimate	*p*	Estimate	*p*	Estimate	*p*	Estimate	*p*	Estimate	*p*	Estimate	*p*
Constant	1.40	0.52	0.04	0.99	0.11	0.96	−0.32	0.86	3.93	0.04	0.22	0.91
Psychological distress (K6)	**0.22**	**<0.01**	**0.15**	**0.03**	**0.14**	**<0.05**	**0.12**	**0.04**	**0.24**	**<0.001**	**0.13**	**0.03**
Suicidality	−0.04	0.61	0.08	0.34	0.11	0.20	**0.20**	**0.01**	0.01	0.86	−0.08	0.25
Exposure to suicide	<0.01	0.84	0.02	0.34	0.02	0.34	**0.04**	**0.02**	0.01	0.65	−0.03	0.12
Entitativity												
Suicide decedent	−0.01	0.59	0.01	0.53	0.01	0.59	<0.01	0.95	0.10	<0.01	**0.05**	**0.01**
Suicide survivors	0.02	0.33	0.02	0.31	0.02	0.43	**0.04**	**0.01**	0.01	0.57	−0.01	0.60
Suicide ideators	−0.01	0.60	−0.03	0.11	−0.04	0.08	0.01	0.41	<0.01	0.84	0.01	0.57
Age	0.08	0.48	0.14	0.20	0.13	0.26	0.09	0.37	−0.07	0.47	0.08	0.42
Male vs. female	−0.05	0.65	0.04	0.69	0.03	0.77	0.09	0.35	−0.06	0.51	0.05	0.58
Heterosexual vs. non-heterosexual	0.27	0.06	0.18	0.21	0.20	0.18	−0.11	0.39	0.02	0.90	0.16	0.19
Major												
Engineering vs. medicine, psychology, or other related disciplines	**0.47**	**<0.01**	0.24	0.10	0.26	0.08	−0.04	0.74	0.19	0.13	**0.32**	**0.01**
Arts vs. medicine, psychology, or other related disciplines	**0.33**	**0.02**	0.19	0.19	0.22	0.15	0.06	0.63	0.16	0.21	0.03	0.79
In a relationship vs. not in a relationship	−0.03	0.77	−0.08	0.48	−0.08	0.49	−0.01	0.91	−0.04	0.69	−0.03	0.78
Parental education level												
Both with tertiary education vs. only one with tertiary education	0.19	0.17	0.12	0.40	0.14	0.32	0.04	0.74	−0.09	0.49	<0.01	0.99
Neither with tertiary education vs. only one with tertiary education	**0.32**	**0.01**	0.18	0.17	0.18	0.18	0.05	0.64	0.03	0.83	0.06	0.61

Note. STOSA, Stigma of Suicide Attempt; STOSSAS, Stigma of Suicide and Suicide Survivor Scale; SOSS, Stigma of Suicide Scale. Bold values indicate *p* < 0.05.

## Data Availability

Data will be stored in a publicly accessible repository and will be available upon publication from the osf.io database (osf.io/94b5e).

## References

[1] Fleischmann A., de Leo D. (2014). The World Health Organization’s report on suicide: A fundamental step in worldwide suicide prevention. Crisis.

[2] Wu C., Jiang G., Duan W. (2018). College students suicide: Current situation and intervention strategies. Heilongjiang Res. High. Educ..

[3] Zhong B.-L., Chiu H.F.K., Conwell Y. (2016). Rates and characteristics of elderly suicide in China, 2013-14. J. Affect. Disord..

[4] Zeng H.J., Zhou G.Y., Yan H.H., Yang X.H., Jin H.M. (2018). Chinese nurses are at high risk for suicide: A review of nurses suicide in China 2007–2016. Arch. Psychiatr. Nurs..

[5] Gunnell D., Chang S.-S., O’Connor R.C., Pirkis J. (2016). Economic recession, unemployment, and suicide. The International Handbook of Suicide Prevention.

[6] Heilbron N., Franklin J.C., Guerry J.D., Prinstein M.J., Nock M.K. (2014). Social and ecological approaches to understanding suicidal behaviors and nonsuicidal self-injury. The Oxford Handbok of Suicide and Self-Injury.

[7] Zhang J., Jing J., Sun W., Wang C. (2011). A sociological analysis of the decline in the suicide rate in China. Soc. Sci. China.

[8] Costanza A., Amerio A., Radomska M., Ambrosetti J., Di Marco S., Prelati M., Aguglia A., Serafini G., Amore M., Bondolfi G. (2020). Suicidality assessment of the elderly with physical illness in the emergency department. Front. Psychiatry.

[9] Corrigan P.W., Sheehan L., Al-Khouja M.A., Lewy S.A., Major D.R., Mead J., Redmon M., Rubey C.T., Weber S. (2017). Making sense of the public stigma of suicide: Factor analyses of its stereotypes, prejudices, and discriminations. Crisis.

[10] Hanschmidt F., Lehnig F., Riedel-Heller S.G., Kersting A. (2016). The stigma of suicide survivorship and related consequences—A systematic review. PLoS ONE.

[11] Corrigan P.W. (2002). The paradox of self-stigma and mental illness. Clin. Psychol. Sci. Pract..

[12] Dueweke A.R., Bridges A.J. (2017). The effects of brief, passive psychoeducation on suicide literacy, stigma, and attitudes toward help-seeking among Latino immigrants living in the United States. Stigma Health.

[13] Han J., Batterham P.J., Calear A.L., Randall R. (2018). Factors influencing professional help-seeking for suicidality. Crisis.

[14] Mackenzie C.S., Visperas A., Ogrodniczuk J.S., Oliffe J.L., Nurmi M.A. (2019). Age and sex differences in self-stigma and public stigma concerning depression and suicide in men. Stigma Health.

[15] Goodwill J.R., Zhou S. (2020). Association between perceived public stigma and suicidal behaviors among college students of color in the U.S. J. Affect. Disord..

[16] WHO (2018). National Suicide Prevention Strategies: Progress, Examples and Indicators.

[17] Lyu J., Wang Y., Shi H., Zhang J. (2018). Early warnings for suicide attempt among Chinese rural population. J. Affect. Disord..

[18] Corrigan P.W., Kerr A., Knudsen L. (2005). The stigma of mental illness: Explanatory models and methods for change. Appl. Prev. Psychol..

[19] Corrigan P.W. (1998). The impact of stigma on severe mental illness. Cogn. Behav. Pract..

[20] Griffiths K.M., Christensen H., Jorm A.F., Evans K., Groves C. (2004). Effect of web-based depression literacy and cognitive-behavioural therapy interventions on stigmatising attitudes to depression. Br. J. Psychiatry J. Ment. Sci..

[21] Scocco P., Castriotta C., Toffol E., Preti A. (2012). Stigma of Suicide Attempt (STOSA) scale and Stigma of Suicide and Suicide Survivor (STOSASS) scale: Two new assessment tools. Psychiatry Res..

[22] Reynders A., Kerkhof A.J.F.M., Molenberghs G., van Audenhove C. (2014). Attitudes and stigma in relation to help-seeking intentions for psychological problems in low and high suicide rate regions. Soc. Psychiatry Psychiatr. Epidemiol..

[23] Muthukrishna M., Schaller M. (2020). Are collectivistic cultures more prone to rapid transformation? Computational models of cross-cultural differences, social network structure, dynamic social influence, and cultural change. Personal. Soc. Psychol. Rev..

[24] Triandis H.C. (1995). Individualism & Collectivism.

[25] Batterham P.J., Calear A.L., Christensen H. (2013). The Stigma of Suicide Scale: Psychometric properties and correlates of the stigma of suicide. Crisis.

[26] Cwik J.C., Till B., Bieda A., Blackwell S.E., Walter C., Teismann T. (2017). Measuring attitudes towards suicide: Preliminary evaluation of an attitude towards suicide scale. Compr. Psychiatry.

[27] Aldalaykeh M., Dalky H., Shahrour G., Rababa M. (2020). Psychometric properties of two Arabic Suicide Scales: Stigma and literacy. Heliyon.

[28] Kawamoto S., Kawashima D., Shiraga K., Kawano K. (2019). Psychometric Consideration of the Japanese Version of the Stigma of Suicide Scale. Jpn. J. Personal..

[29] Han J., Batterham P.J., Calear A.L., Wu Y., Shou Y., van Spijker B.A.J. (2017). Translation and validation of the Chinese versions of the Suicidal Ideation Attributes Scale, Stigma of Suicide Scale, and Literacy of Suicide Scale. Death Stud..

[30] Calear A.L., Batterham P.J., Christensen H. (2014). Predictors of help-seeking for suicidal ideation in the community: Risks and opportunities for public suicide prevention campaigns. Psychiatry Res..

[31] Kearns M., Muldoon O.T., Msetfi R.M., Surgenor P.W.G. (2015). Understanding help-seeking amongst university students: The role of group identity, stigma, and exposure to suicide and help-seeking. Front. Psychol..

[32] Scocco P., Preti A., Totaro S., Corrigan P.W., Castriotta C., Bianchera F., Facchini S., Ferrari A., Guadagnini M., Toffol E. (2019). Stigma, grief and depressive symptoms in help-seeking people bereaved through suicide. J. Affect. Disord..

[33] Scocco P., Preti A., Totaro S., Ferrari A., Toffol E. (2017). Stigma and psychological distress in suicide survivors. J. Psychosom. Res..

[34] Sheehan L., Dubke R., Corrigan P.W. (2017). The specificity of public stigma: A comparison of suicide and depression-related stigma. Psychiatry Res..

[35] Cerel J., Brown M.M., Maple M., Singleton M., van de Venne J., Moore M., Flaherty C. (2019). How many people are exposed to suicide? Not six. Suicide Life-Threat. Behav..

[36] Pitman A.L., Osborn D.P.J., Rantell K., King M.B. (2016). Bereavement by suicide as a risk factor for suicide attempt: A cross-sectional national UK-wide study of 3432 young bereaved adults. BMJ Open.

[37] Till B., Wild T.A., Arendt F., Scherr S., Niederkrotenthaler T. (2018). Associations of tabloid newspaper use with endorsement of suicide myths, suicide-related knowledge, and stigmatizing attitudes toward suicidal individuals. Crisis.

[38] Batterham P.J., Calear A.L., Christensen H. (2013). Correlates of suicide stigma and suicide literacy in the community. Suicide Life-Threat. Behav..

[39] Silverman M.M., O’Connor R.C., Pirkis J. (2016). Challenges to defining and classifying suicide and suicidal behaviors. The International Handbook of Suicide Prevention.

[40] Oexle N., Waldmann T., Staiger T., Xu Z., Rusch N. (2018). Mental illness stigma and suicidality: The role of public and individual stigma. Epidemiol. Psychiatr. Sci..

[41] Xu Z., Muller M., Heekeren K., Theodoridou A., Metzler S., Dvorsky D., Oexle N., Walitza S., Rossler W., Rusch N. (2016). Pathways between stigma and suicidal ideation among people at risk of psychosis. Schizophr. Res..

[42] Scocco P., Toffol E., Preti A. (2016). Psychological distress increases perceived stigma toward attempted suicide among those with a history of past attempted suicide. J. Nerv. Ment. Dis..

[43] Campbell D.T. (1958). Common fate, similarity, and other indices of the status of aggregates of persons as social entities. Behav. Sci..

[44] Lickel B., Hamilton D.L., Wieczorkowska G., Lewis A., Sherman S.J., Uhles A.N. (2000). Varieties of groups and the perception of group entitativity. J. Personal. Soc. Psychol..

[45] Blanchard A.L., Caudill L.E., Walker L.S. (2018). Developing an entitativity measure and distinguishing it from antecedents and outcomes within online and face-to-face groups. Group Process. Intergroup Relat..

[46] Roets A., Van Hiel A. (2011). The role of need for closure in essentialist entitativity beliefs and prejudice: An epistemic needs approach to racial categorization. Br. J. Soc. Psychol..

[47] Haslam N., Ernst D. (2002). Essentialist beliefs about mental disorders. J. Soc. Clin. Psychol..

[48] Haslam N., Levy S.R. (2006). Essentialist beliefs about homosexuality: Structure and implications for prejudice. Personal. Soc. Psychol. Bull..

[49] Hamilton D.L., Chen J.M., Way N., Kramer R.M., Leonardelli G., Livingston R. (2011). Dynamic aspects of entitativity: From group perception to social interaction. Social Cognition, Social Identity, and Intergroup Relations: A Festschrift in Honor of Marilynn B. Brewer.

[50] Hamilton D.L., Chen J.M., Ko D.M., Winczewski L., Banerji I., Thurston J.A. (2015). Sowing the seeds of stereotypes: Spontaneous inferences about groups. J. Personal. Soc. Psychol..

[51] McConnell A.R., Sherman S.J., Hamilton D.L. (1994). On-line and memory-based aspects of individual and group target judgments. J. Personal. Soc. Psychol..

[52] McConnell A.R., Sherman S.J., Hamilton D.L. (1997). Target entitativity: Implications for information processing about individual and group targets. J. Personal. Soc. Psychol..

[53] Crawford M.T., Sherman S.J., Hamilton D.L. (2002). Perceived entitativity, stereotype formation, and the interchangeability of group members. J. Personal. Soc. Psychol..

[54] Gaertner L., Schopler J. (1998). Perceived ingroup entitativity and intergroup bias: An interconnection of self and others. Eur. J. Soc. Psychol..

[55] Kende A., McGarty C. (2019). A model for predicting prejudice and stigma expression by understanding target perceptions: The effects of visibility, politicization, responsibility, and entitativity. Eur. J. Soc. Psychol..

[56] Rüsch N., Corrigan P.W., Wassel A., Michaels P., Olschewski M., Wilkniss S., Batia K. (2009). Ingroup perception and responses to stigma among persons with mental illness. Acta Psychiatr. Scand..

[57] Alessandri G., Vecchione M., Fagnani C., Bentler P.M., Barbaranelli C., Medda E., Nisticò L., Stazi M.A., Caprara G.V. (2010). Much more than model fitting? Evidence for the heritability of method effect associated with positively worded items of the Life Orientation Test revised. Struct. Eq. Modeling Multidiscip. J..

[58] Lindwall M., Barkoukis V., Grano C., Lucidi F., Raudsepp L., Liukkonen J., Thøgersen-Ntoumani C. (2012). Method effects: The problem with negatively versus positively keyed items. J. Personal. Assess..

[59] Woods C.M. (2006). Careless responding to reverse-worded items: Implications for confirmatory factor analysis. J. Psychopathol. Behav. Assess..

[60] Weijters B., Baumgartner H., Schillewaert N. (2013). Reversed item bias: An integrative model. Psychol. Methods.

[61] Podsakoff P.M., MacKenzie S.B., Podsakoff N.P. (2012). Sources of method bias in social science research and recommendations on how to control it. Annu. Rev. Psychol..

[62] Wu Y., Zuo B., Wen F., Yan L. (2017). Rosenberg Self-Esteem Scale: Method effects, factorial structure and scale invariance across migrant child and urban child populations in China. J. Personal. Assess..

[63] Eid M., Geiser C., Koch T. (2016). Measuring method effects: From traditional to design-oriented approaches. Curr. Dir. Psychol. Sci..

[64] Calear A.L., Batterham P.J., Sunderland M., Carragher N. (2019). Development and validation of static and adaptive screeners to assess suicidal thoughts and behavior. Suicide Life Threat. Behav..

[65] Kessler R.C., Andrews G., Colpe L.J., Hiripi E., Mroczek D.K., Normand S.L.T., Walters E.E., Zaslavsky A.M. (2002). Short screening scales to monitor population prevalences and trends in non-specific psychological distress. Psychol. Med..

[66] Kessler R.C., Green J.G., Gruber M.J., Sampson N.A., Bromet E., Cuitan M., Furukawa T.A., Gureje O., Hinkov H., Hu C.-Y. (2010). Screening for serious mental illness in the general population with the K6 screening scale: Results from the WHO World Mental Health (WMH) survey initiative. Int. J. Methods Psychiatr. Res..

[67] Holmes E.P., Corrigan P.W., Williams P., Canar J., Kubiak M.A. (1999). Changing attitudes about schizoprenia. Schizophr. Bull..

[68] Han J. (2017). Professional Help Seeking for Suicidal Ideation among Chinese and Australian University Students. Ph.D. Thesis.

[69] Meade A.W., Craig S.B. (2012). Identifying careless responses in survey data. Psychol. Methods.

[70] Hu L.-t., Bentler P.M. (1998). Fit indices in covariance structure modeling: Sensitivity to underparameterized model misspecification. Psychol. Methods.

[71] Hu L.-t., Bentler P.M. (1999). Cutoff criteria for fit indexes in covariance structure analysis: Conventional criteria versus new alternatives. Struct. Eq. Modeling A Multidiscip. J..

[72] Marsh H.W., Balla J.R., McDonald R.P. (1988). Goodness-of-fit indexes in confirmatory factor analysis: The effect of sample size. Psychol. Bull..

[73] Sivo S.A., Fan X., Witta E.L., Willse J.T. (2006). The search for “optimal” cutoff properties: Fit index criteria in structural equation modeling. J. Exp. Educ..

[74] Carpiniello B., Pinna F. (2017). The reciprocal relationship between suicidality and stigma. Front. Psychiatry.

[75] Wu F. (2009). Life for Justice: A Cultural Interpretation of Suicide Phenomenon in a County in North China.

[76] Han J., Batterham P.J., Calear A.L., Wu Y., Xue J., van Spijker B. (2018). Development and pilot evaluation of an online psychoeducational program for suicide prevention among university students: A randomised controlled trial. Internet Interv..

[77] Berardelli I., Erbuto D., Rogante E., Sarubbi S., Lester D., Pompili M. (2020). Making sense of the unique pain of survivors: A Psychoeducational approach for suicide bereavement. Front. Psychol..

